# Influence of Clinical Factors and Magnification Correction on Normal Thickness Profiles of Macular Retinal Layers Using Optical Coherence Tomography

**DOI:** 10.1371/journal.pone.0147782

**Published:** 2016-01-27

**Authors:** Tomomi Higashide, Shinji Ohkubo, Masanori Hangai, Yasuki Ito, Noriaki Shimada, Kyoko Ohno-Matsui, Hiroko Terasaki, Kazuhisa Sugiyama, Paul Chew, Kenneth K. W. Li, Nagahisa Yoshimura

**Affiliations:** 1 Department of Ophthalmology and Visual Science, Kanazawa University Graduate School of Medical Science, Kanazawa, Japan; 2 Department of Ophthalmology, Saitama Medical University, Saitama, Japan; 3 Department of Ophthalmology, Nagoya University Graduate School of Medicine, Nagoya, Japan; 4 Department of Ophthalmology and Visual Science, Tokyo Medical and Dental University, Tokyo, Japan; 5 Department of Ophthalmology, National University Hospital, National University Health System, Singapore, Singapore; 6 Department of Ophthalmology, United Christian Hospital and Tseung Kwan O Hospital, Hong Kong, China; 7 Department of Ophthalmology and Visual Sciences, Kyoto University Graduate School of Medicine, Kyoto, Japan; University of Florida, UNITED STATES

## Abstract

**Purpose:**

To identify the factors which significantly contribute to the thickness variabilities in macular retinal layers measured by optical coherence tomography with or without magnification correction of analytical areas in normal subjects.

**Methods:**

The thickness of retinal layers {retinal nerve fiber layer (RNFL), ganglion cell layer plus inner plexiform layer (GCLIPL), RNFL plus GCLIPL (ganglion cell complex, GCC), total retina, total retina minus GCC (outer retina)} were measured by macular scans (RS-3000, NIDEK) in 202 eyes of 202 normal Asian subjects aged 20 to 60 years. The analytical areas were defined by three concentric circles (1-, 3- and 6-mm nominal diameters) with or without magnification correction. For each layer thickness, a semipartial correlation (sr) was calculated for explanatory variables including age, gender, axial length, corneal curvature, and signal strength index.

**Results:**

Outer retinal thickness was significantly thinner in females than in males (sr^2^, 0.07 to 0.13) regardless of analytical areas or magnification correction. Without magnification correction, axial length had a significant positive sr with RNFL (sr^2^, 0.12 to 0.33) and a negative sr with GCLIPL (sr^2^, 0.22 to 0.31), GCC (sr^2^, 0.03 to 0.17), total retina (sr^2^, 0.07 to 0.17) and outer retina (sr^2^, 0.16 to 0.29) in multiple analytical areas. The significant sr in RNFL, GCLIPL and GCC became mostly insignificant following magnification correction.

**Conclusions:**

The strong correlation between the thickness of inner retinal layers and axial length appeared to result from magnification effects. Outer retinal thickness may differ by gender and axial length independently of magnification correction.

## Introduction

Evolution of optical coherence tomography (OCT) technologies has made *in vivo* thickness measurements of retinal layers an indispensable tool for the diagnosis and management of various diseases involving the macula. In particular, inner retinal layers, i.e. ganglion cell complex (GCC), retinal nerve fiber layer (RNFL), and the ganglion cell layer plus inner plexiform layer (GCLIPL), are the primary targets to quantitate retinal ganglion cell (RGC) loss in the macula owing to the improved thickness measurements by spectral-domain OCT [1]. Furthermore, given that the macula is where more than half of RGCs reside to serve as output neurons for the central vision [2], *in vivo* quantification of RGCs in the macula provides vital information to elucidate the structure-function relationship in glaucoma and other diseases [3–6].

Determinants of macular layer thickness in normal subjects have been extensively studied in order to establish a normative database for improving diagnostic accuracy [7–10] and also to reveal normal anatomical nature of the macula by utilizing data from population-based studies [11–14]. Various factors including age [7,9–22], gender[7–14,16–20,22–26], ethnicity [7–9,25,26], eye laterality [8,9], axial length [9–14,16–18,20,22,23,26–30], refractive error [11,12,14,17,18,20,22,23,26–28,30], corneal curvature [13,14,18], and signal strength [9,12,18,31] have been selected as potential candidates that may influence retinal layer thickness in the macula. However, only a few studies have conducted a multivariate regression analysis to determine the relationship between various clinical factors and the thickness of multiple retinal layers [10,17,18,22]. Although dependencies on age and ethnicity are relatively well characterized and are incorporated into the normative databases of commercially available OCT devices, the clinical relevance of other factors remain to be established.

A number of studies reported the significant effects of axial length on the retinal layer thickness in the macula [9,11,12–14,16,17,20,22,26–29,32]. Some authors speculated that retinal thinning in myopic eyes may result from mechanical stretching of the sclera along with axial elongation [13,14,17,23,27]. However, another possibility is artificial retinal thinning due to ocular magnification effects on the analytical area, which is inherent to OCT scanning [9–11,17,23,28,29,31]. In this regard, a negative correlation between RNFL thickness, measured by a peripapillary 12º circular scan, and axial length was eliminated by correcting the magnification effects on the size of scan circle [32–35]. However, thickness changes by magnification correction of the macular analytical area were only reported for total retinal thickness in one study [31]. Furthermore, the influence of magnification correction on the correlation between various clinical factors and the thickness of multiple retinal layers has not yet been reported. In terms of the clinical significance, inner retinal thinning associated with a long axial length may have a significant impact on the diagnosis of early glaucoma in highly myopic eyes due to an increase in false positives [36,37].

The purpose of this study was to comprehensively investigate the relationship between clinical factors and the thickness of multiple retinal layers in the macula measured by OCT in normal subjects and to identify the factors that significantly contribute to the variability in thickness. Furthermore, the influence of magnification correction of the analytical area on the contribution was also examined. To this end, we enrolled eyes with a broad range of axial length.

## Subjects and Methods

### Study design

This was a prospective, cross-sectional, multicenter study conducted in six clinical centers; Kyoto University (Japan), Nagoya University (Japan), Kanazawa University (Japan), the National University Health System (Singapore), Tokyo Medical and Dental University (Japan) and Tseung Kwan O Hospital (Hong Kong). The study adhered to the tenets of the Declaration of Helsinki and approval of the Institutional Review Board was obtained at each participating center. Asian adult subjects were enrolled in each site. Written informed consent was obtained from each subject.

### Protocols of ocular examination

The study subjects underwent a complete ocular examination: best corrected visual acuity (BCVA), refractive status by autorefractometry, intraocular pressure (IOP) using Goldmann applanation tonometry, anterior segment examination by slit lamp biomicroscopy, fundus examination by ophthalmoscopy and slit lamp biomicroscopy, and fundus photography. Ocular biometry, i.e. corneal curvature using autokeratometry and axial length using partial coherence interferometry (IOLMaster; Carl Zeiss Meditec, Inc., Dublin, CA, USA), was also measured. Standard automated perimetry (SAP) was performed using a Humphrey field analyzer (Carl Zeiss Meditec) with the 24–2 Swedish Interactive Threshold Algorithm. Macular scans by spectral-domain OCT (RS-3000, Nidek, Gamagori, Aichi, Japan), which consisted of 128 vertical B-scans, each of which had 512 A-scans, were performed by well-trained examiners at each institution over a 30×30° square area, corresponding to 9×9 mm square area in the Gullstrand model eye. Only high quality images with the highest possible signal strength index (SSI) achievable, no centration errors, and no motion artifacts were used.

### Exclusion criteria

Subjects with medical history of diabetes, hypertension, central nervous system diseases, use of medications such as systemic steroids and anti-cancer drugs, ocular surgeries including corneal refractive surgery, intraocular surgeries and retinal photocoagulation were excluded. Exclusion criteria for ocular examination were BCVA <1.0, IOP >21 mmHg, glaucomatous abnormalities in reliable SAP results (fixation loss ≤20%, false-negative error ≤15%, false-positive error ≤33%) meeting the Anderson’s criteria in which one of the following was present: having a cluster of 3 or more non-edge points with P < 5% and at least one point with P < 1% in the pattern deviation probability plot, pattern standard deviation of less than 5%, or a glaucoma hemifield test result outside normal limits. Eyes with shallow anterior chamber or media opacity affecting the fundus imaging, low quality OCT images, fundus abnormalities such as glaucoma, diabetic retinopathy, retinal detachment, macular degeneration, retinitis pigmentosa, retinal vein occlusion, inferior staphyloma associated with tilted disc syndrome, scleral ridge temporal to the optic disc, peripapillary intrachoroidal cavitation or diffuse chorioretinal atrophy were also excluded.

In order to study the influence of magnification correction of the analytical area, we aimed to enroll eyes with a broad range of axial length. Accordingly, exclusion criteria were set for spherical equivalent refractive error (>+3.0 diopters) and axial length (>30 mm). Because of the difficulty of enrollment of elderly, highly myopic eyes without any pathological changes in the macula, we did not include subjects older than 60 years in this study. Even distribution of subjects by gender was aimed in each decade of age. When both eyes were eligible for inclusion, one eye was randomly chosen.

### Analyses of the thickness of macular retinal layers

The thickness of retinal layers {RNFL, GCLIPL, RNFL plus GCLIPL (GCC), total retina, total retina minus GCC (outer retina)} in the macular scans was measured using automatic segmentation software. The segmentation of each layer in all B-scan images was inspected and corrected manually if necessary.

**Fig 1:**
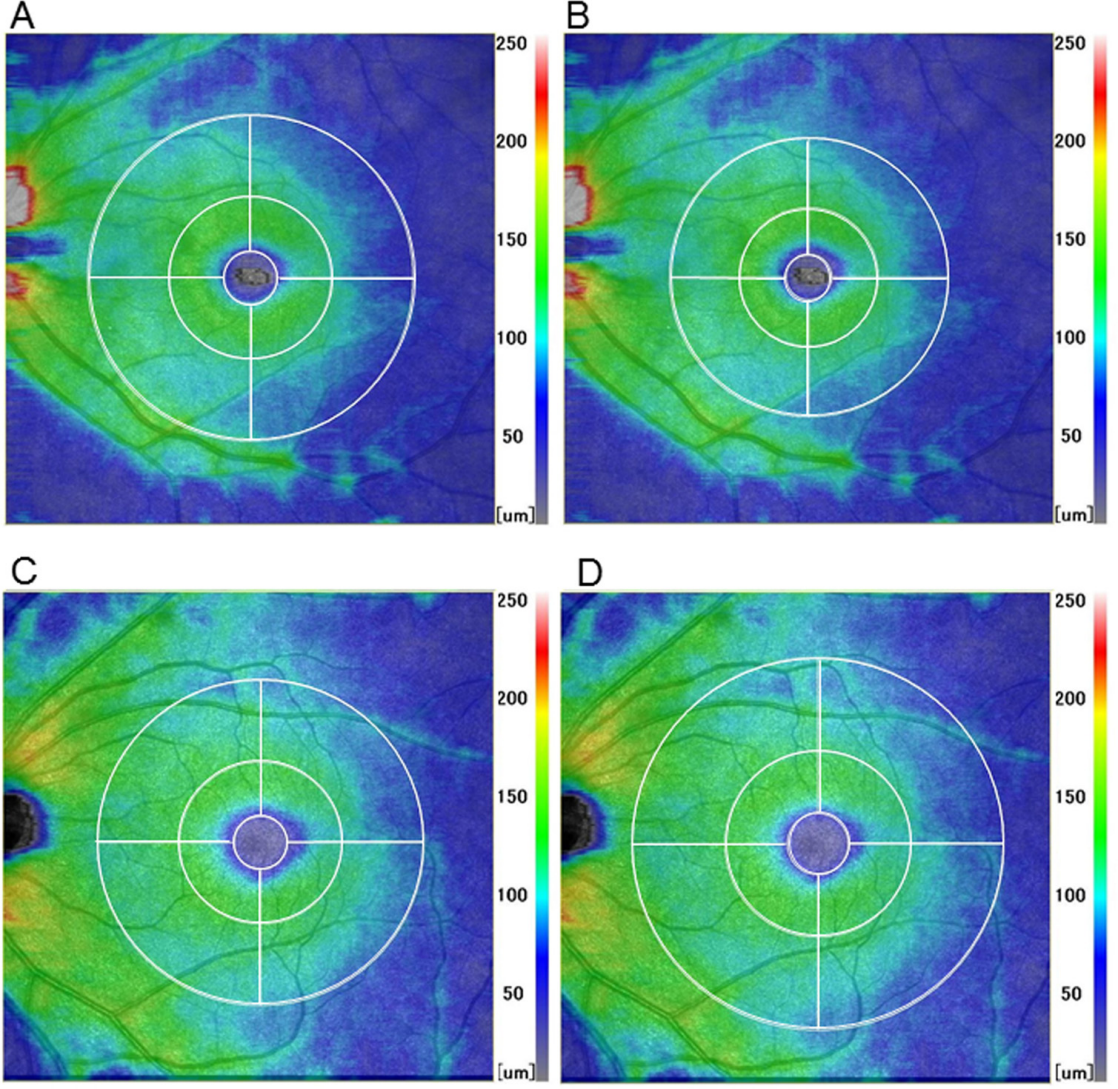
Thickness maps of the ganglion cell complex showing the effects of magnification correction on the analytical area. A highly myopic eye with an axial length of 28.41 mm (A, B) and a hyperopic eye with an axial length of 21.71 mm (C, D). The 30×30°square scan area is supposed to be 9×9 mm square area for any eye when magnification correction is not considered (A, C). In this situation, the apparent sizes of the analytical area on the recorded images, which is defined by three concentric circles (1-, 3- and 6-mm nominal diameters), are identical regardless of the axial length (A, C). However, the scan area corresponded to a 10.5x10.5 mm (B) or 7.9x7.9 mm (D) square area after magnification correction in eyes with a long or short axial length, respectively. The sizes of three concentric circles were rescaled according to the dimension of the ‘magnification-corrected’ scan area, and defined as the ‘magnification-corrected’ analytical area (B, D). The analytical area became relatively smaller or larger compared to the scan area by magnification correction in eyes with a long or short axial length, respectively (B, D).

The analytical area was defined by three concentric circles, 3.3°-, 10°- and 20°-diameters, which correspond to 1-, 3- and 6-mm diameters in the Gullstrand model eye with an axial length of 24.38 mm, respectively. The inner (1–3 mm) and outer (3–6 mm) rings were further divided into four quadrants: inferior nasal, inferior temporal, superior temporal, and superior nasal. The 30×30° square scan area is supposed to be a nominal 9×9 mm square area for all eyes when magnification correction is not considered. A modified Littmann’s formula (Bennett’s formula) was used to correct the ocular magnification effect associated with OCT scans [38]. By entering the data of axial length into the formula, a magnification-corrected analytical area was determined for each scan. For example, the scan area corresponded to a 10.5x10.5 mm square area after magnification correction in an eye with an axial length of 28.41 mm. The sizes of three concentric circles were, then, rescaled according to the dimension of the ‘magnification-corrected’ scan area, and this was defined as the ‘magnification-corrected’ analytical area (Fig 1). Left eye data were converted to a right eye format.

### Statistical analysis

Average thickness of retinal layers in each analytical area with or without magnification correction was compared using a paired t-test. Equality of variance between the thickness with and without magnification correction was examined by F-test. P-values were adjusted using Bonferroni correction given the 12 analytical areas to be compared for each retinal layer. Correlations between two explanatory variables were examined using a Spearman’s rank correlation coefficient.

For each retinal layer thickness in each analytical area as an objective variable, a semipartial correlation coefficient (sr) was determined in the multiple linear regression model with seven explanatory variables: age, gender, eye laterality, axial length, spherical equivalent, corneal curvature, and SSI. A significant sr means that a significant amount of variance of the objective variable is explained by an explanatory variable independently of other explanatory variables. A sr^2^ (sr squared) indicates the proportion of total variance in the objective variable uniquely accounted for by an explanatory variable controlling for other explanatory variables. A sr was also determined for the correlation of explanatory variables with retinal layer thickness in the ‘magnification-corrected’ analytical area.

Regarding the issue of multicollinearity, when two or more explanatory variables were highly correlated each other and had high variance inflation factors (VIF >5), a variable with the smallest partial correlation coefficient controlling for the highly correlated variables was removed from the set of explanatory variables to be entered in the regression analysis, and VIFs were recalculated. A partial residual plot was employed to visualize the relationship between a given explanatory variable and the objective variable with the effect of the other explanatory variables in the model. Statistical analyses were performed with SPSS software (IBM SPSS Statistics 20, IBM Corp., New York, USA) and STATA software (STATA/MP version 14.0; StataCorp, TX, USA). For all analyses, a P-value of <0.05 was considered statistically significant.

## Results

The characteristics of subjects including demographic and ocular biometric data are shown in Table 1. Male subjects (35.3, 20.7–58.2 years; median, range) were significantly younger than female subjects (40.5, 20.3–59.9 years; P = 0.047, Mann-Whitney test). Correlations between these variables evaluated by Spearman’s rank correlation coefficient are shown in Table 2. Axial length had significant correlations with other variables, especially with spherical equivalent refractive error (rs, -0.89).

**Table 1:** Characteristics of the subjects including demographic and ocular biometric data.

Number of subjects/ eyes	202/ 202
Age (years), mean ± SD (range)	38.4 ± 10.8 (20.3–59.9)
Gender (female/ male)	95/ 107
Eye laterality (right/ left eye)	101/ 101
Axial length (mm), mean ± SD (range)	25.8 ± 1.7 (21.5–28.8)
Spherical equivalent refractive error (diopters), mean ± SD (range)	-5.1 ± 3.9 (1.75 - -15.5)
Corneal curvature (mm), mean ± SD (range)	7.75 ± 0.24 (7.16–8.60)
Signal strength index, mean ± SD (range)	8.4 ± 1.1 (6–10)

SD = standard deviation. Data are shown as mean ± standard deviation.

**Table 2:** Correlation between two explanatory variables by Spearman’s rank correlation coefficient.

Variables		Age (years)	Gender	Eye laterality	Axial length	Spherical equivalent refractive error	Corneal curvature
Gender (female vs. male)	rs	0.14					
	P-value	0.046					
Eye laterality (left vs. right)	rs	0.01	0.03				
	P-value	0.94	0.67				
Axial length (mm)	rs	-0.20	-0.21	0.03			
	P-value	0.004	0.003	0.68			
Spherical equivalent refractive error (diopters)	rs	0.18	0.08	-0.03	-0.89		
	P-value	0.009	0.27	0.72	<0.0001		
Corneal curvature (mm)	rs	-0.21	-0.15	-0.03	0.29	0.04	
	P-value	0.003	0.036	0.68	<0.0001	0.55	
Signal strength index	rs	-0.08	0.10	0.12	-0.36	0.34	-0.16
	P-value	0.23	0.14	0.10	<0.0001	<0.0001	0.02

rs = Spearman’s rank correlation coefficient.

First, the influence of the magnification correction of the analytical area on the thickness of retinal layers was examined. Average thickness of retinal layers in each analytical area with or without magnification correction was compared (Table 3). Except for areas including some quadrants of outer ring of RNFL and inner ring of GCLIPL, average thickness significantly changed after magnification correction of the analytical area. RNFL and inner ring of GCC were significantly thinner in the ‘magnification-corrected’ analytical area. On the other hand, the outer ring of GCLIPL and GCC and all areas of the outer and total retina except for the center became significantly thicker after magnification correction. The thickness variance became significantly smaller after magnification correction in the outer ring of GCLIPL.

**Table 3:** Average thickness of retinal layers in each analytical area with or without magnification correction.

Retinal layers	RNFL		GCLIPL		GCC		Total retina		Outer retina	
Mag. Corr.	-	+	-	+	-	+	-	+	-	+
Total area	34.8 ± 3.0*	33.9 ± 2.5*	68.7 ± 5.6*	70.6 ± 4.7*	103.5 ± 6.5*	104.5 ± 6.2*	303.0 ± 12.2*	304.7 ± 11.4*	198.4 ± 8.6*	200.2 ± 7.7*	
Center	5.6 ± 5.2*	5.2 ± 4.9*	41.8 ± 7.4*	39.7 ± 7.9*	47.4 ± 8.2*	44.9 ± 7.3*	264.0 ± 16.2	263.9 ± 16.4	219.5 ± 11.2*	219.0 ± 11.4*	
IR (total)	25.8 ± 3.2*	25.1 ± 2.6*	92.3 ± 6.5	92.3 ±6.6	118.1 ± 7.3*	117.4 ± 7.2*	336.3 ± 13.4*	337.6 ± 13.3*	219.2 ± 9.3*	220.2 ± 8.9*	
OR (total)	38.6 ± 3.4*	37.5 ± 2.9*	62.7 ± 6.1*^†^	65.3 ± 4.7*^†^	101.2 ± 7.2*	102.9 ± 6.6*	294.6 ± 12.6*	296.5 ± 11.5*	191.4 ± 8.7*	193.6 ± 7.6*	
IR (IN)	28.1 ± 3.8*	27.0 ± 3.1*	92.3 ± 6.5	92.3 ±6.7	120.4 ± 7.6*	119.4 ± 7.6*	338.1 ± 14.0*	339.7 ± 13.8*	219.1 ± 10.0*	220.3 ± 9.5*	
IR (IT)	24.2 ± 3.8*	23.7 ± 3.4*	92.1 ±7.1	92.3 ± 7.1	116.3 ± 7.5*	116.0 ± 7.5*	331.7 ± 13.5*	332.9 ± 13.4*	215.9 ± 9.4*	216.9 ± 9.0*	
IR (ST)	24.2 ± 3.2*	23.7 ± 2.8*	91.1 ± 6.8*	91.4 ± 6.8*	115.3 ± 7.2	115.1 ± 7.1	333.5 ± 13.2*	334.5 ± 13.1*	218.6 ± 9.3*	219.4 ± 8.9*	
IR (SN)	26.9 ± 3.7*	25.8 ± 2.9*	93.5 ± 6.8	93.4 ± 6.9	120.4 ± 7.9*	119.2 ± 7.7*	342.1 ± 14.2*	343.5 ± 14.1*	223.3 ± 9.7*	224.2 ± 9.2*	
OR (IN)	49.4 ± 6.0*^†^	47.1 ± 4.8*^†^	61.8 ± 6.2*^†^	64.5 ± 4.7*^†^	111.3 ± 8.6	111.6 ± 7.9	299.8 ± 14.2*	302.0 ± 13.1*	188.2 ± 9.5*	190.4 ± 8.2*	
OR (IT)	28.7 ± 2.8	28.6 ± 2.7	61.5 ± 6.5*^†^	64.2 ± 5.3*^†^	90.2 ± 7.1*	92.8 ± 6.3*	280.6 ± 12.3*	282.3 ± 11.4*	187.3 ± 8.6*	189.5 ± 7.7*	
OR (ST)	27.9 ± 2.7	27.9 ± 2.7	62.1 ± 6.5*	64.6 ± 5.7*	89.9 ± 7.5*	92.6 ± 6.9*	286.7 ± 12.7*	288.1 ± 11.9*	193.6 ± 8.5*	195.6 ± 7.7*	
OR (SN)	48.3 ± 5.0*	46.5 ± 4.2*	65.2 ± 6.5*^†^	68.0 ± 5.0*^†^	113.5 ± 8.1*	114.5 ± 7.7*	311.3 ± 13.8*	313.4 ± 12.6*	196.6 ± 9.6*	198.9 ± 8.3*	

RNFL = retinal nerve fiber layer, GCLIPL = ganglion cell layer plus inner plexiform layer, GCC = ganglion cell complex, Mag. Corr. = magnification correction, IR = inner ring, OR = outer ring, IN = inferior nasal, IT = inferior temporal, ST = superior temporal, SN = superior nasal.

*P <0.05 after Bonferroni correction (paired t-test).

^†^P<0.05 after Bonferroni correction (F-test of equality of variances). Data are shown as mean ± standard deviation (μm).

**Fig 2:**
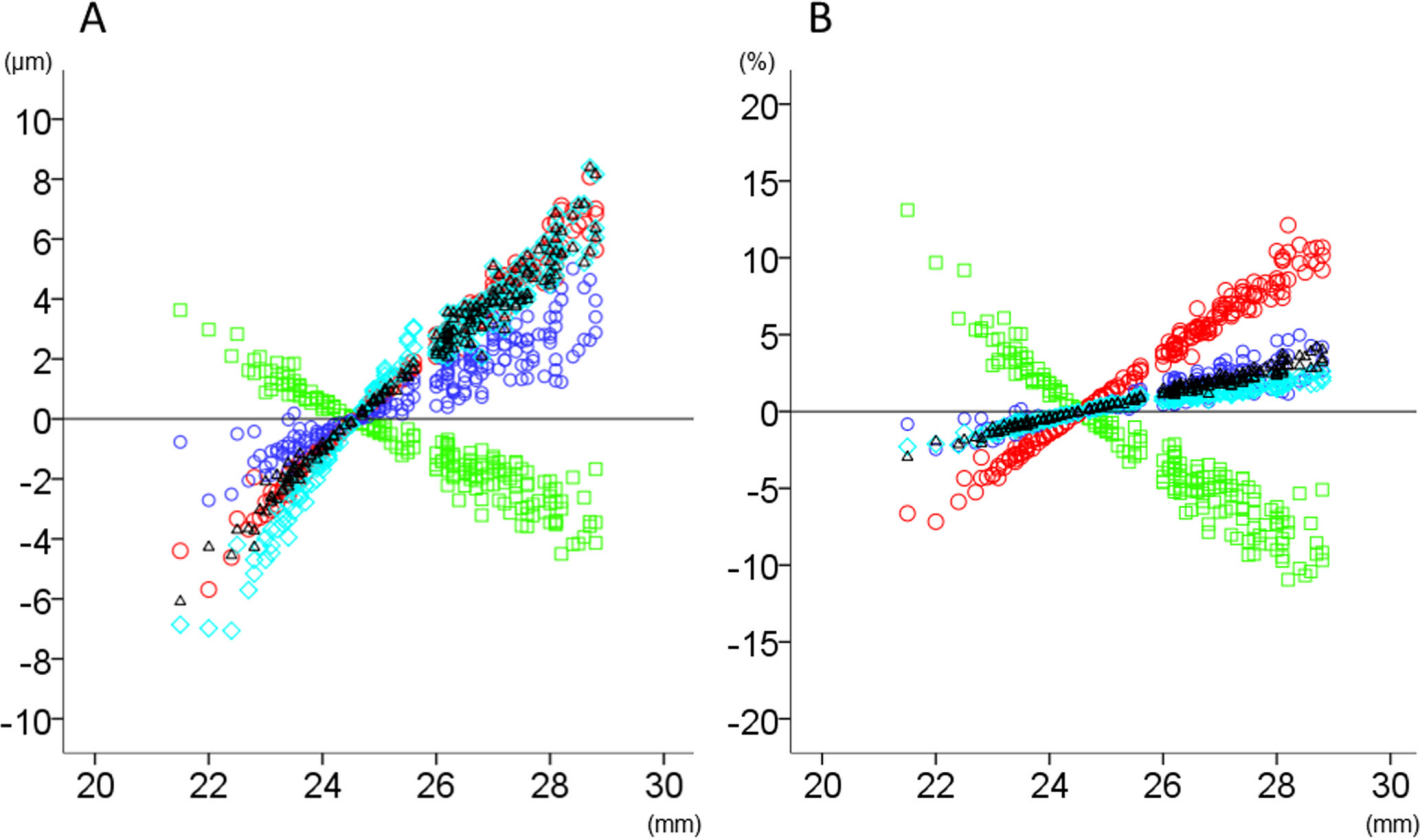
The relationship between axial length and thickness changes of retinal layers in the total analytical area after magnification correction. The thickness changes (μm; A) and relative thickness changes compared to the magnification-uncorrected thickness (%; B) after magnification correction were plotted against axial length. Green squares: retinal nerve fiber layer, Red circles: ganglion cell layer plus inner plexiform layer, Blue circles: ganglion cell complex, Light blue diamonds: total retina, Black triangles: outer retina.

**Fig 3:**
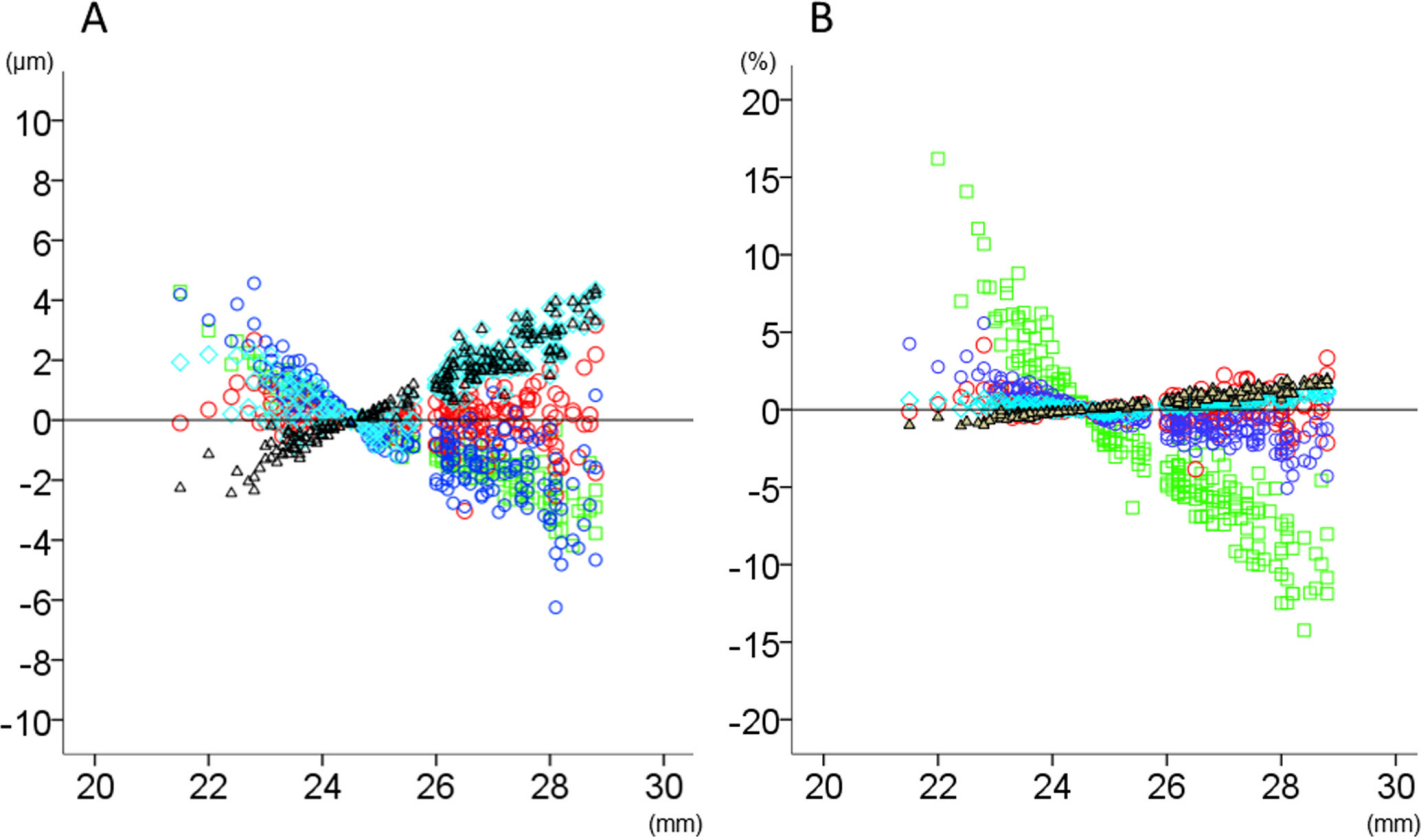
The relationship between axial length and thickness changes of retinal layers in the inner ring of analytical area after magnification correction. The thickness changes (μm; A) and relative thickness changes compared to the magnification-uncorrected thickness (%; B) after magnification correction were plotted against axial length. Green squares: retinal nerve fiber layer, Red circles: ganglion cell layer plus inner plexiform layer, Blue circles: ganglion cell complex, Light blue diamonds: total retina, Black triangles: outer retina.

**Fig 4:**
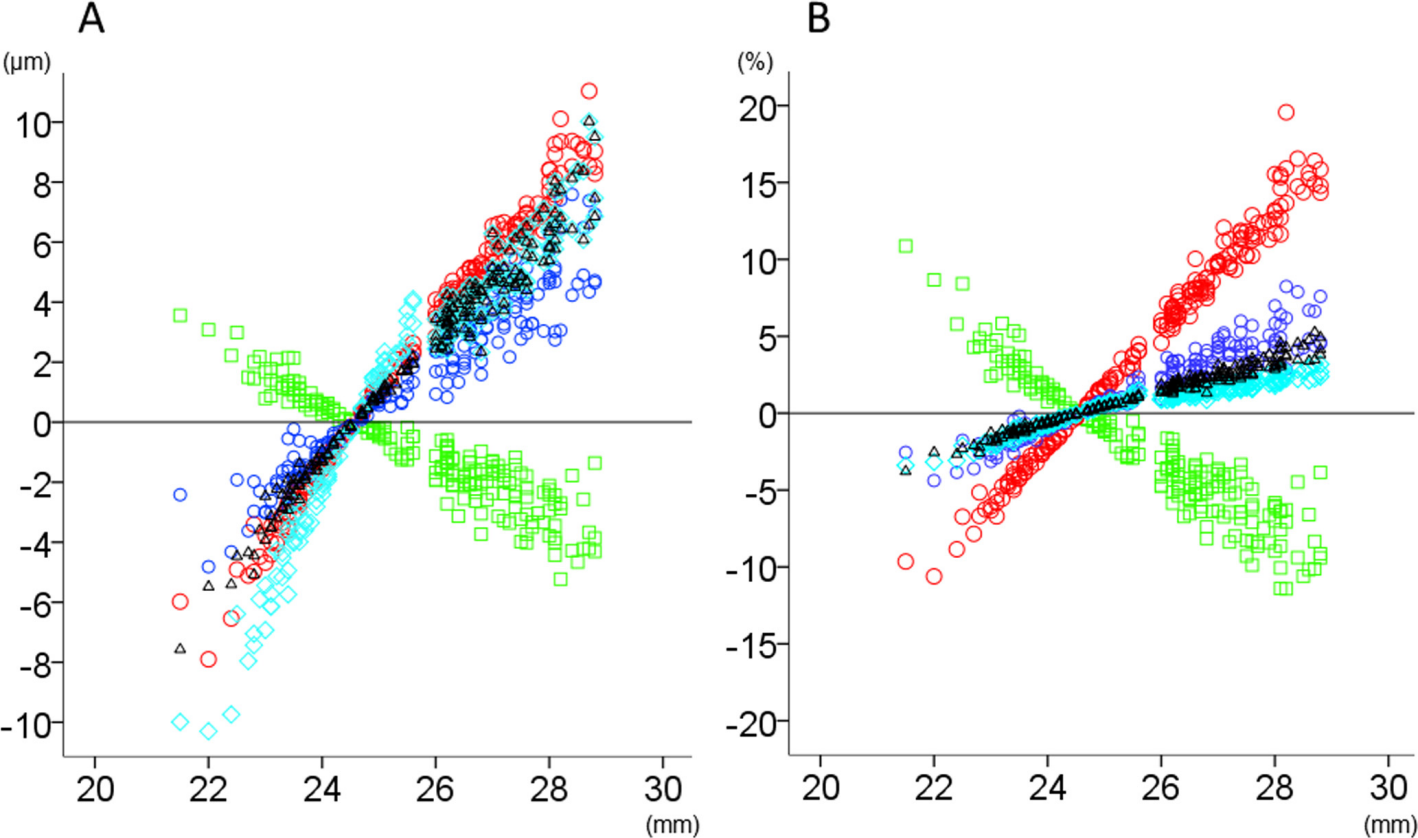
The relationship between axial length and thickness changes of retinal layers in the outer ring of analytical area after magnification correction. The thickness changes (μm; A) and relative thickness changes compared to the magnification-uncorrected thickness (%; B) after magnification correction were plotted against axial length. Green squares: retinal nerve fiber layer, Red circles: ganglion cell layer plus inner plexiform layer, Blue circles: ganglion cell complex, Light blue diamonds: total retina, Black triangles: outer retina.

The relationship between axial length and thickness changes (μm or percentage relative to the magnification-uncorrected thickness) after magnification correction in total area and inner and outer rings is shown in Figs 2–4. The magnitude of thickness changes in each retinal layer tended to increase as the axial length deviated from 24.38 mm. RNFL was the only retinal layer which showed thickness changes correlated negatively with axial length in all three areas. Other retinal layers had smaller thickness changes than RNFL, when expressed as percentage of the magnification-uncorrected thickness, whereas apparent positive slopes were observed in total area and the outer ring. The thickness in the inner ring showed relatively smaller changes than the outer ring, which was most obvious in GCLIPL. The inner retinal layers had more thickness percentage changes than total or outer retina, especially in the inner ring of RNFL and in the outer ring of GCLIPL and RNFL.

Influences of demographic and ocular biometric variables on each retinal layer thickness per analytical area were examined by multiple linear regression analysis. Among seven exploratory variables examined, axial length and spherical equivalent refractive error, which were highly correlated each other (rs, -0.89), had high VIFs (12.0 and 11.0, respectively). Given that partial correlation coefficients adjusted for each other were smaller in refractive error than in axial length for almost all the layer thicknesses, refractive error was omitted as an explanatory variable, which reduced the VIFs of all the other variables to <2. With these six variables entered into the multiple regression models, all variables except corneal curvature then had a significant sr with at least one retinal layer (S1–S5 Tables). Age, eye laterality, and signal strength index had a significant sr in some analytical areas of several layers although sr^2^ values were small (≤0.03).

**Fig 5:**
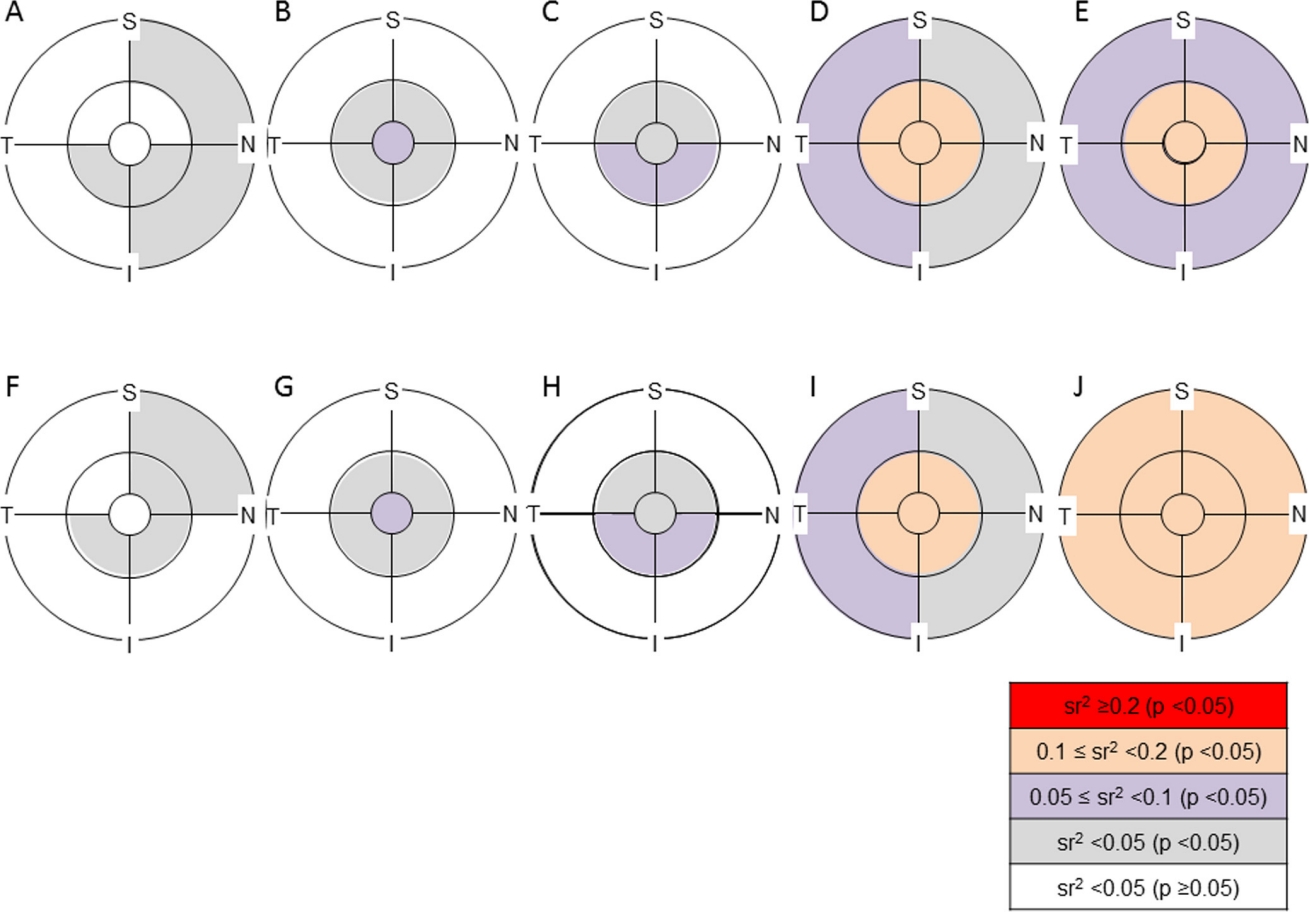
Semipartial correlation of gender with the thickness of retinal layers in each analytical area with or without magnification correction. The significance of the semipartial correlation coefficient (sr) and magnitude of the sr squared (sr^2^) are color coded in each analytical area. S = superior, N = nasal, I = inferior, T = temporal. A, F: retinal nerve fiber layer, B, G: ganglion cell layer plus inner plexiform layer, C, H: ganglion cell complex, D, I: total retina, E, J: outer retina. A—E: ‘magnification-uncorrected’ analytical area, F—J: ‘magnification-corrected’ analytical area. A significant sr was found in the inner ring of inner retinal layers and in all the analytical areas of outer and total retina. The sr^2^ was relatively large in total and outer retina compared to inner retinal layers. The magnification correction of analytical areas did not cause remarkable changes in the magnitude of sr^2^ values compared to the sr of axial length with the thickness of retinal layers (Fig 6).

Gender and axial length had a significant sr in all layers. In terms of gender, the significant sr was mostly negative, indicating that the thickness controlling for other variables was significantly smaller in females than in males (S1–S5 Tables, Fig 5). The results of simple between-gender comparisons of retinal layer thickness (S6 Table) were similar to those of sr. A significant negative sr was found in the inner ring of the inner retinal layers (sr^2^, 0.01 to 0.07) and in all the analytical areas of outer and total retina (sr^2^, 0.05 to 0.13). The magnification correction of analytical areas made the sr^2^ values slightly larger in many of the analytical areas. The sr^2^ was especially large in the ‘magnification-corrected’ analytical areas of the outer retina where sr^2^ values were all ≥0.1.

**Fig 6:**
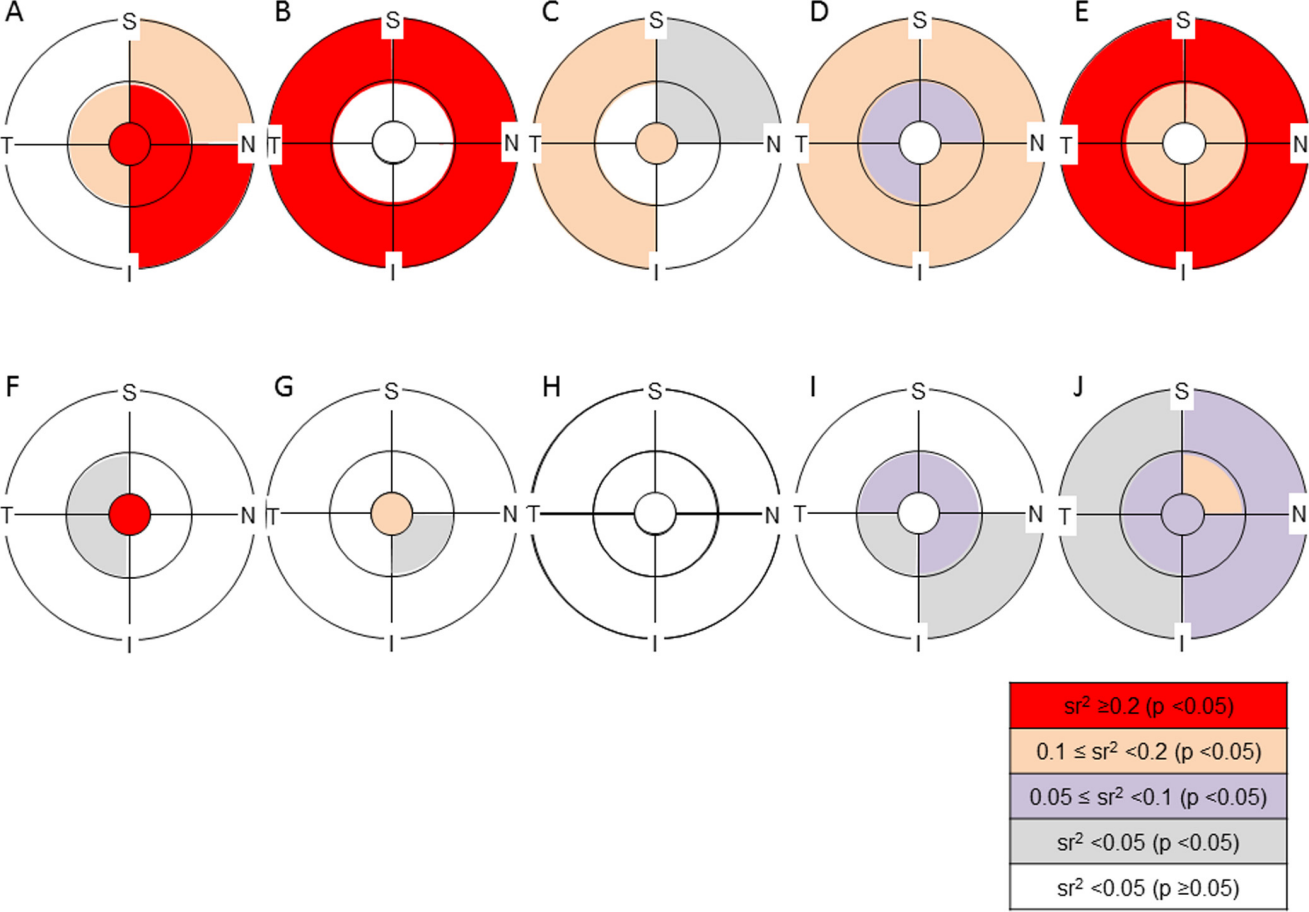
Semipartial correlation of axial length with the thickness of retinal layers in each analytical area with or without magnification correction. The significance of the semipartial correlation coefficient (sr) and magnitude of the sr squared (sr^2^) are color coded in each analytical area. S = superior, N = nasal, I = inferior, T = temporal. A, F: retinal nerve fiber layer (RNFL), B, G: ganglion cell layer plus inner plexiform layer (GCLIPL), C, H: ganglion cell complex (GCC), D, I: total retina, E, J: outer retina. A—E: ‘magnification-uncorrected’ analytical area, F—J: ‘magnification-corrected’ analytical area. A large sr^2^ (≥0.2) was observed in RNFL, GCLIPL and outer retina in ‘magnification-uncorrected’ analytical areas. After magnification correction, most of the sr in the inner retinal layers, especially in GCC, became insignificant. In contrast, the sr were still significant in most of the analytical areas of the outer and total retina after magnification correction.

Regarding the sr of axial length in ‘magnification-uncorrected’ analytical areas, a significant positive sr (sr^2^ ≥0.1) was observed in the RNFL except for the temporal part of the outer ring, whereas a significant negative sr (sr^2^ ≥0.2) was found in the outer ring of GCLIPL (S1–S5 Tables, Fig 6). As the sum of RNFL and GCLIPL, the negative sr in the outer ring was less significant in GCC than in GCLIPL. After magnification correction, most of the sr in the inner retina layers, especially in the GCC, became insignificant. In contrast, for the outer and total retinas, all analytical areas except for the center had a significant negative sr (sr^2^ ≥0.05), which were still significant in most of the analytical areas after the magnification correction although sr^2^ values became smaller.

**Fig 7:**
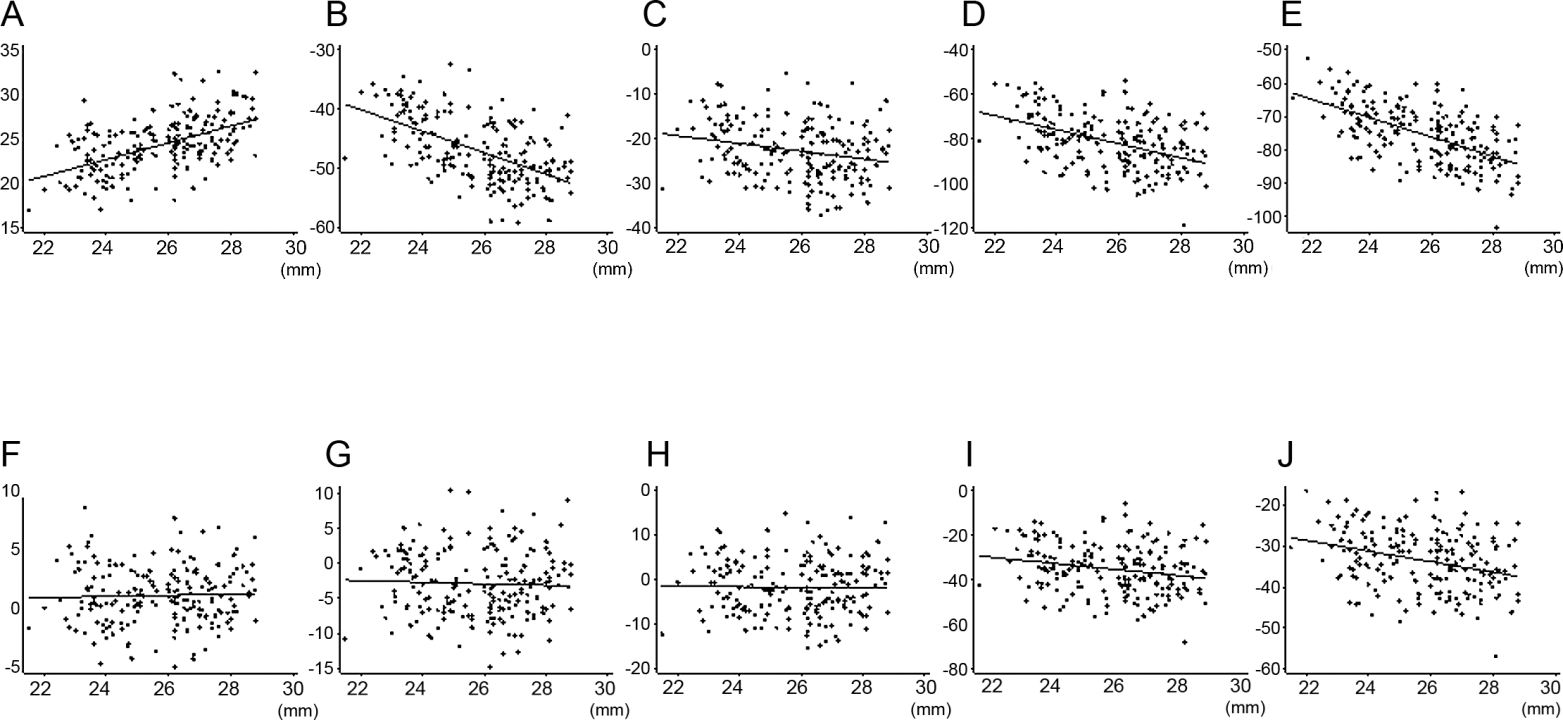
Partial residual plots showing the relationship between axial length and the average thickness of each retinal layer in total analytical area controlling for the effects of other five explanatory variables. Component plus residual was plotted against axial length for each retinal layer. A, F: retinal nerve fiber layer, B, G: ganglion cell layer plus inner plexiform layer, C, H: ganglion cell complex, D, I: total retina, E, J: outer retina. A—E: magnification uncorrected analytical area, F—J: magnification corrected analytical area. The other five explanatory variables = age, gender, eye laterality, corneal curvature, and signal strength index.

Fig 7 shows partial residual plots to illustrate the relationship between axial length and the average thickness of total analytical area in each retinal layer controlling for the effects of the other five explanatory variables. RNFL showed a positive correlation (sr, 0.46) with axial length, while GCLIPL (sr, -0.48) and GCC (sr, -0.20) showed negative correlations, but only without magnification correction. The negative correlations of total (sr, -0.39) and outer retina (sr, -0.51) without magnification correction became weaker, but were still significant after magnification correction (sr, -0.18 and -0.25, respectively).

## Discussion

Without magnification correction, a negative correlation between axial length and retinal thickness in the macula has been reported for full thickness [13,14,16,17,20,22,26,28] and also for GCC [22,29] and GCLIPL [9,12,29,30], while RNFL thickness of the macular area had a positive correlation with axial length [29]. Our results, which corroborate the earlier reports, may be explained by the normal thickness profile of each retinal layer determined by spectral-domain OCT [10,39]. For example, GCLIPL has an annular-shaped thickest portion in the pericentral region, which makes the layer thicker in the inner ring than in the outer ring. Therefore, the average GCLIPL thickness in the outer ring becomes gradually thinner in eyes with longer axial length than the model eye due to widening of the analytical area when the magnification effect is not corrected. The fact that the magnification correction almost nullified the dependency of thickness in the inner retinal layers on the axial length indicates that axial elongation may not significantly affect inner retinal thickness in the macula. In contrast, axial length retained significantly negative contributions to outer retinal thickness even after magnification correction. This implies that the thickness of the outer part of the retina has a true axial length dependency which may partly be associated with scleral stretching by axial length elongation.

Gender also had a significant contribution to macular retinal thickness. The results of thinner macula in females than in males agree with previous reports [9–14,16,17,19,20,22–26]. In this study subjects, males were significantly younger than females although even distribution of subjects by gender was aimed. The effect of age imbalance on gender difference was adjusted in multivariate regression analysis for determining a sr of gender. Given that the sr^2^ values for the outer retina were larger than those for the inner retina in all analytical areas, gender may have a greater influence on the outer retina than on the inner retina. Ooto *et al*. reported that the inner nuclear layer, outer plexiform layer and outer nuclear layer were the retinal layers where the thickness was significantly greater in males than in females [10]. Therefore, gender differences in the outer retina or total retina observed in this study may be attributable to these layers. The increase in sr^2^ values of gender for the outer ring of outer retina after magnification correction suggested that the gender differences in the thickness of these layers may be attenuated outside of the nominal 6-mm diameter circle. The attenuation of gender differences may be accounted for by thinning of the outer nuclear layer from the center of the macula towards the periphery. The clinical significance of the thickness variabilities by gender or axial length can be compared with the measurement reproducibility by OCT. Spectral-domain OCT devices have a high reproducibility of thickness measurements in macular scans. The coefficient of variations (CVs) were <1.5% in total retina [40,41], <1.5% in GCC [42], <1% in GCLIPL [29,42], and <2.5% in RNFL [29,42] when measured in normal subjects. Using RS-3000, Nakanishi *et al*. reported that the average CVs of thickness in GCC within the nominal 9-mm diameter circle were <2% in highly-myopic eyes with or without glaucoma [37]. Meanwhile, gender and axial length had ≥10% contribution (i.e. sr^2^ ≥0.1) to the thickness variance in many of the analytical areas of multiple retinal layers. For example, given that the mean, standard deviation, variance and sr^2^ of axial length for GCLIPL thickness in the ‘magnification-uncorrected’ total area were 68.7 μm, 5.6 μm, 31.4 μm^2^, and 0.23, respectively, the variance that was uniquely attributable to axial length was 7.2 μm^2^, which is larger than the reproducibility variance corresponding to a CV of 3% (4.2 μm^2^). On the other hand, given that the mean, standard deviation, variance and sr^2^ of gender for the outer retinal thickness in the ‘magnification-corrected’ total area were 200.2 μm, 7.7 μm, 59.3 μm^2^, and 0.13, respectively, the variance that was uniquely attributable to gender was 7.7 μm^2^, which is smaller than the reproducibility variance corresponding to a CV of 1.5% (9.0 μm^2^). Sr^2^ values ≥0.03 or ≥0.1 were needed for GCLIPL or GCC and RNFL to exceed the reproducibility variance corresponding to the CV of normal subjects in all ‘magnification-uncorrected’ analytical areas, while sr^2^ values ≥0.2 were required for total or outer retina in all analytical areas regardless of magnification correction. Sr^2^ of gender was ≤0.07 for inner retinal layers and was ≤0.15 for total and outer retina. Among them, only the inner ring of GCLIPL had comparably large sr^2^ values to the reproducibility variance of normal subjects regardless of magnification correction. In contrast, sr^2^ values of axial length were large enough to exceed the reproducibility variance in multiple ‘magnification-uncorrected’ analytical areas of all retinal layers except for total retina. Therefore, axial length, under the condition of ‘magnification-uncorrected’ analytical areas, and gender for GCLIPL appeared to have significant clinical impacts on the layer thickness in comparison with OCT measurement reproducibility.

Furthermore, regarding the magnification correction, as shown in Fig 2, a considerable proportion of eyes had thickness changes larger than the CVs of measurement reproducibility of each retinal layer in normal subjects (i.e. 1 to 2.5%). In particular, the thickness change may be ≥5% in the outer ring of GCLIPL in eyes with an axial length >26 mm. Furthermore, the statistically significant reduction of thickness variance by magnification correction was observed in the outer ring of GCLIPL. Thus, magnification correction is also clinically significant for the thickness variability, especially in the inner retinal layers, and the results indicated that magnification correction may be useful for the construction and application of normative database involving the outer ring of GCLIPL.

Our study has several limitations. We did not include subjects older than 60 years because of the difficulty in recruiting elderly, highly myopic subjects with a strictly normal fundus. This may be one reason why we failed to find a significant contribution of age to retinal layer thickness. In addition, we used a modified Littmann’s formula to correct the ocular magnification effect associated with OCT scans. Although this method was proven to be equally accurate to more detailed calculations using additional ocular biometric parameters [43], under- or over-corrections may occur in eyes where ocular dimensions deviated from the assumption in the formula. Regarding racial differences in normal thickness profiles of macular retinal layers, applicability of our results to non-Asians needs to be determined in further studies.

In conclusion, we determined the unique contribution of various factors to thickness variabilities in macular retinal layers measured by OCT with or without magnification correction of analytical areas in normal subjects. Gender and axial length appeared to significantly contribute to the thickness variance of all retinal layers in the macula. A significant correlation between the thickness of the inner retinal layers and axial length appeared to result mostly from magnification effects. Outer retinal thickness may differ by gender and axial length independently of magnification correction. The significance of the clinical factors, especially axial length and magnification correction, for the diagnosis of various diseases involving the macula needs to be further elucidated.

## Supporting Information

S1 Table Semipartial correlation of various factors with the thickness of the retinal nerve fiber layer in each analytical area. (DOCX)

S2 Table Semipartial correlation of various factors with the thickness of the ganglion cell layer plus inner plexiform layer in each analytical area. (DOCX)

S3 Table Semipartial correlation of various factors with the thickness of the ganglion cell complex in each analytical area. (DOCX)

S4 Table Semipartial correlation of various factors with the thickness of the total retina in each analytical area. (DOCX)

S5 Table Semipartial correlation of various factors with the thickness of the outer retina in each analytical area (DOCX)

S6 Table Between-gender differences in the thickness of retinal layers in each analytical area. (DOCX)

## Abbreviations

OCT: optical coherence tomography
GCC: ganglion cell complex
RNFL: retinal nerve fiber layer
GCLIPL: ganglion cell layer plus inner plexiform layer
RGC: retinal ganglion cell
BCVA: best corrected visual acuity
IOP: intraocular pressure
SAP: standard automated perimetry
SSI: signal strength index
sr: semipartial correlation coefficient
VIF: variance inflation factors
CV: coefficient of variations
