# Supplementary material for: Influence of Clinical Factors and Magnification Correction on Normal Thickness Profiles of Macular Retinal Layers Using Optical Coherence Tomography
Source: PLoS One. 2016 Jan 27;11(1):e0147782. doi: 10.1371/journal.pone.0147782 (PMC4729678; doi:10.1371/journal.pone.0147782)
Supplement: S2 Table — (DOCX) [file pone.0147782.s002.docx]

| S2 Table. Semipartial correlation of various factors with the thickness of the ganglion cell layer plus inner plexiform layer in each analytical area | | | | | | | | | | | | | | | | | | | |
| --- | --- | --- | --- | --- | --- | --- | --- | --- | --- | --- | --- | --- | --- | --- | --- | --- | --- | --- | --- |
| Factors | | Age | | | Gender | | | Eye laterality | | | Axial length | | | Corneal curvature | | | Signal strength index | | |
|  | | sr | sr^2^ | p value | sr | sr^2^ | p value | sr | sr^2^ | p value | sr | sr^2^ | p value | sr | sr^2^ | p value | sr | sr^2^ | p value |
| Magnification-uncorrected | total area | -0.07 | 0.01 | 0.25 | -0.11 | 0.01 | 0.07 | 0.05 | 0.00 | 0.36 | -0.48 | 0.23 | <0.0001 | 0.01 | 0.00 | 0.90 | 0.08 | 0.01 | 0.19 |
|  | Center | 0.03 | 0.00 | 0.72 | -0.24 | 0.06 | 0.0001 | -0.03 | 0.00 | 0.69 | -0.02 | 0.00 | 0.73 | 0.05 | 0.00 | 0.49 | 0.11 | 0.01 | 0.11 |
|  | inner ring (total) | -0.13 | 0.02 | 0.05 | -0.18 | 0.03 | 0.01 | 0.03 | 0.00 | 0.62 | -0.12 | 0.01 | 0.08 | -0.01 | 0.00 | 0.92 | 0.14 | 0.02 | 0.04 |
|  | outer ring (total) | -0.04 | 0.00 | 0.44 | -0.06 | 0.00 | 0.25 | 0.06 | 0.00 | 0.29 | -0.55 | 0.31 | <0.0001 | 0.01 | 0.00 | 0.87 | 0.05 | 0.00 | 0.40 |
|  | inner ring (IN) | -0.14 | 0.02 | 0.04 | -0.19 | 0.04 | 0.01 | 0.05 | 0.00 | 0.43 | -0.13 | 0.02 | 0.06 | 0.01 | 0.00 | 0.87 | 0.12 | 0.01 | 0.08 |
|  | inner ring (IT) | -0.14 | 0.02 | 0.04 | -0.19 | 0.04 | 0.01 | 0.00 | 0.00 | 0.96 | -0.13 | 0.02 | 0.06 | -0.02 | 0.00 | 0.74 | 0.16 | 0.03 | 0.02 |
|  | inner ring (ST) | -0.13 | 0.02 | 0.06 | -0.17 | 0.03 | 0.01 | 0.02 | 0.00 | 0.82 | -0.11 | 0.01 | 0.10 | -0.01 | 0.00 | 0.83 | 0.14 | 0.02 | 0.04 |
|  | inner ring (SN) | -0.10 | 0.01 | 0.17 | -0.15 | 0.02 | 0.04 | 0.07 | 0.00 | 0.35 | -0.08 | 0.01 | 0.23 | 0.00 | 0.00 | 0.98 | 0.13 | 0.02 | 0.07 |
|  | outer ring (IN) | -0.05 | 0.00 | 0.35 | 0.02 | 0.00 | 0.77 | 0.06 | 0.00 | 0.24 | -0.56 | 0.31 | <0.0001 | -0.03 | 0.00 | 0.59 | 0.01 | 0.00 | 0.84 |
|  | outer ring (IT) | -0.06 | 0.00 | 0.30 | -0.10 | 0.01 | 0.09 | 0.02 | 0.00 | 0.79 | -0.51 | 0.26 | <0.0001 | -0.01 | 0.00 | 0.88 | 0.04 | 0.00 | 0.46 |
|  | outer ring (ST) | -0.06 | 0.00 | 0.35 | -0.09 | 0.01 | 0.12 | 0.05 | 0.00 | 0.43 | -0.47 | 0.22 | <0.0001 | 0.03 | 0.00 | 0.64 | 0.06 | 0.00 | 0.37 |
|  | outer ring (SN) | 0.01 | 0.00 | 0.91 | -0.06 | 0.00 | 0.27 | 0.09 | 0.01 | 0.09 | -0.56 | 0.31 | <0.0001 | 0.04 | 0.00 | 0.43 | 0.07 | 0.00 | 0.22 |
| Magnification-corrected | total area | -0.08 | 0.01 | 0.28 | -0.14 | 0.02 | 0.06 | 0.06 | 0.00 | 0.39 | -0.04 | 0.00 | 0.62 | 0.01 | 0.00 | 0.94 | 0.10 | 0.01 | 0.17 |
|  | Center | 0.02 | 0.00 | 0.81 | -0.21 | 0.05 | 0.0001 | -0.02 | 0.00 | 0.75 | -0.35 | 0.12 | <0.0001 | 0.04 | 0.00 | 0.55 | 0.10 | 0.01 | 0.13 |
|  | inner ring (total) | -0.12 | 0.02 | 0.08 | -0.19 | 0.04 | 0.01 | 0.02 | 0.00 | 0.76 | -0.12 | 0.02 | 0.07 | -0.01 | 0.00 | 0.88 | 0.15 | 0.02 | 0.03 |
|  | outer ring (total) | -0.05 | 0.00 | 0.46 | -0.09 | 0.01 | 0.21 | 0.07 | 0.01 | 0.30 | 0.03 | 0.00 | 0.72 | 0.01 | 0.00 | 0.90 | 0.06 | 0.00 | 0.37 |
|  | inner ring (IN) | -0.12 | 0.01 | 0.08 | -0.20 | 0.04 | 0.004 | 0.05 | 0.00 | 0.50 | -0.14 | 0.02 | 0.04 | 0.01 | 0.00 | 0.90 | 0.13 | 0.02 | 0.05 |
|  | inner ring (IT) | -0.13 | 0.02 | 0.05 | -0.20 | 0.04 | 0.003 | -0.02 | 0.00 | 0.83 | -0.12 | 0.02 | 0.07 | -0.03 | 0.00 | 0.71 | 0.17 | 0.03 | 0.02 |
|  | inner ring (ST) | -0.12 | 0.02 | 0.07 | -0.18 | 0.03 | 0.01 | 0.00 | 0.00 | 0.97 | -0.09 | 0.01 | 0.20 | -0.02 | 0.00 | 0.81 | 0.15 | 0.02 | 0.03 |
|  | inner ring (SN) | -0.09 | 0.01 | 0.19 | -0.15 | 0.02 | 0.03 | 0.05 | 0.00 | 0.49 | -0.12 | 0.01 | 0.08 | -0.01 | 0.00 | 0.93 | 0.12 | 0.02 | 0.08 |
|  | outer ring (IN) | -0.07 | 0.01 | 0.33 | 0.00 | 0.00 | 0.97 | 0.07 | 0.01 | 0.33 | -0.03 | 0.00 | 0.72 | -0.04 | 0.00 | 0.54 | 0.00 | 0.00 | 0.99 |
|  | outer ring (IT) | -0.07 | 0.00 | 0.35 | -0.12 | 0.02 | 0.08 | 0.01 | 0.00 | 0.84 | 0.05 | 0.00 | 0.49 | -0.01 | 0.00 | 0.90 | 0.06 | 0.00 | 0.37 |
|  | outer ring (ST) | -0.07 | 0.00 | 0.35 | -0.12 | 0.01 | 0.10 | 0.06 | 0.00 | 0.39 | 0.06 | 0.00 | 0.43 | 0.03 | 0.00 | 0.64 | 0.08 | 0.01 | 0.28 |
|  | outer ring (SN) | 0.01 | 0.00 | 0.89 | -0.08 | 0.01 | 0.29 | 0.13 | 0.02 | 0.07 | 0.01 | 0.00 | 0.94 | 0.05 | 0.00 | 0.51 | 0.09 | 0.01 | 0.22 |
| sr = semipartial correlation coefficient, sr^2^ = semipartial correlation squared, IN = inferior nasal, IT = inferior temporal, ST = superior temporal, SN = superior nasal. | | | | | | | | | | | | | | | | | | | |
