# Supplementary material for: Influence of Clinical Factors and Magnification Correction on Normal Thickness Profiles of Macular Retinal Layers Using Optical Coherence Tomography
Source: PLoS One. 2016 Jan 27;11(1):e0147782. doi: 10.1371/journal.pone.0147782 (PMC4729678; doi:10.1371/journal.pone.0147782)
Supplement: S3 Table — (DOCX) [file pone.0147782.s003.docx]

| S3 Table. Semipartial correlation of various factors with the thickness of the ganglion cell complex in each analytical area | | | | | | | | | | | | | | | | | | | |
| --- | --- | --- | --- | --- | --- | --- | --- | --- | --- | --- | --- | --- | --- | --- | --- | --- | --- | --- | --- |
| Factors | | Age | | | Gender) | | | Eye laterality | | | Axial length | | | Corneal curvature | | | Signal strength index | | |
|  | | sr | sr^2^ | p value | sr | sr^2^ | p value | sr | sr^2^ | p value | sr | sr^2^ | p value | sr | sr^2^ | p value | sr | sr^2^ | p value |
| Magnification-uncorrected | total area | -0.07 | 0.01 | 0.28 | -0.04 | 0.00 | 0.52 | 0.08 | 0.01 | 0.23 | -0.20 | 0.04 | 0.004 | 0.01 | 0.00 | 0.85 | 0.10 | 0.01 | 0.13 |
|  | Center | 0.02 | 0.00 | 0.77 | -0.21 | 0.04 | 0.001 | 0.00 | 0.00 | 0.95 | 0.34 | 0.11 | <0.0001 | 0.01 | 0.00 | 0.87 | 0.04 | 0.00 | 0.49 |
|  | inner ring (total) | -0.12 | 0.01 | 0.09 | -0.21 | 0.05 | 0.002 | 0.04 | 0.00 | 0.52 | 0.11 | 0.01 | 0.12 | 0.02 | 0.00 | 0.82 | 0.10 | 0.01 | 0.13 |
|  | outer ring (total) | -0.06 | 0.00 | 0.40 | 0.02 | 0.00 | 0.77 | 0.09 | 0.01 | 0.19 | -0.29 | 0.08 | <0.0001 | 0.01 | 0.00 | 0.87 | 0.09 | 0.01 | 0.16 |
|  | inner ring (IN) | -0.12 | 0.01 | 0.08 | -0.22 | 0.05 | 0.001 | 0.10 | 0.01 | 0.14 | 0.12 | 0.01 | 0.07 | 0.01 | 0.00 | 0.87 | 0.07 | 0.01 | 0.30 |
|  | inner ring (IT) | -0.10 | 0.01 | 0.16 | -0.24 | 0.06 | 0.0004 | 0.00 | 0.00 | 0.99 | 0.06 | 0.00 | 0.41 | 0.01 | 0.00 | 0.83 | 0.10 | 0.01 | 0.16 |
|  | inner ring (ST) | -0.12 | 0.02 | 0.07 | -0.19 | 0.03 | 0.01 | 0.01 | 0.00 | 0.88 | 0.05 | 0.00 | 0.44 | 0.03 | 0.00 | 0.69 | 0.12 | 0.01 | 0.09 |
|  | inner ring (SN) | -0.11 | 0.01 | 0.10 | -0.17 | 0.03 | 0.01 | 0.05 | 0.00 | 0.42 | 0.17 | 0.03 | 0.01 | 0.01 | 0.00 | 0.90 | 0.12 | 0.01 | 0.09 |
|  | outer ring (IN) | -0.06 | 0.00 | 0.37 | 0.11 | 0.01 | 0.12 | 0.16 | 0.02 | 0.02 | -0.10 | 0.01 | 0.15 | -0.02 | 0.00 | 0.75 | 0.10 | 0.01 | 0.15 |
|  | outer ring (IT) | -0.04 | 0.00 | 0.54 | -0.08 | 0.01 | 0.20 | 0.00 | 0.00 | 0.96 | -0.42 | 0.17 | <0.0001 | 0.01 | 0.00 | 0.85 | 0.05 | 0.00 | 0.39 |
|  | outer ring (ST) | -0.06 | 0.00 | 0.32 | -0.05 | 0.00 | 0.40 | 0.06 | 0.00 | 0.34 | -0.41 | 0.17 | <0.0001 | 0.05 | 0.00 | 0.46 | 0.07 | 0.01 | 0.28 |
|  | outer ring (SN) | -0.04 | 0.00 | 0.55 | 0.08 | 0.01 | 0.27 | 0.09 | 0.01 | 0.19 | -0.18 | 0.03 | 0.01 | 0.01 | 0.00 | 0.90 | 0.12 | 0.01 | 0.08 |
| Magnification-corrected | total area | -0.08 | 0.01 | 0.28 | -0.07 | 0.01 | 0.33 | 0.08 | 0.01 | 0.27 | -0.02 | 0.00 | 0.82 | 0.01 | 0.00 | 0.94 | 0.11 | 0.01 | 0.13 |
|  | center | 0.02 | 0.00 | 0.82 | -0.21 | 0.04 | 0.003 | 0.01 | 0.00 | 0.90 | -0.03 | 0.00 | 0.64 | 0.02 | 0.00 | 0.77 | 0.04 | 0.00 | 0.54 |
|  | inner ring (total) | -0.11 | 0.01 | 0.10 | -0.23 | 0.05 | 0.0008 | 0.03 | 0.00 | 0.65 | -0.06 | 0.00 | 0.40 | -0.01 | 0.00 | 0.93 | 0.11 | 0.01 | 0.13 |
|  | outer ring (total) | -0.06 | 0.00 | 0.39 | 0.00 | 0.00 | 0.97 | 0.09 | 0.01 | 0.22 | 0.00 | 0.00 | 1.00 | 0.01 | 0.00 | 0.92 | 0.10 | 0.01 | 0.17 |
|  | inner ring (IN) | -0.11 | 0.01 | 0.10 | -0.25 | 0.06 | 0.0003 | 0.09 | 0.01 | 0.20 | -0.10 | 0.01 | 0.16 | -0.01 | 0.00 | 0.86 | 0.08 | 0.01 | 0.24 |
|  | inner ring (IT) | -0.09 | 0.01 | 0.19 | -0.26 | 0.07 | 0.0002 | -0.01 | 0.00 | 0.91 | -0.04 | 0.00 | 0.54 | 0.00 | 0.00 | 0.98 | 0.09 | 0.01 | 0.18 |
|  | inner ring (ST) | -0.11 | 0.01 | 0.10 | -0.20 | 0.04 | 0.01 | -0.01 | 0.00 | 0.94 | -0.02 | 0.00 | 0.74 | 0.01 | 0.00 | 0.89 | 0.12 | 0.02 | 0.08 |
|  | inner ring (SN) | -0.11 | 0.01 | 0.10 | -0.19 | 0.04 | 0.01 | 0.04 | 0.00 | 0.54 | -0.06 | 0.00 | 0.38 | -0.02 | 0.00 | 0.77 | 0.11 | 0.01 | 0.10 |
|  | outer ring (IN) | -0.06 | 0.00 | 0.38 | 0.08 | 0.01 | 0.26 | 0.14 | 0.02 | 0.04 | -0.08 | 0.01 | 0.25 | -0.04 | 0.00 | 0.58 | 0.07 | 0.01 | 0.33 |
|  | outer ring (IT) | -0.05 | 0.00 | 0.51 | -0.10 | 0.01 | 0.16 | -0.01 | 0.00 | 0.88 | 0.07 | 0.01 | 0.32 | 0.01 | 0.00 | 0.85 | 0.07 | 0.01 | 0.34 |
|  | outer ring (ST) | -0.07 | 0.01 | 0.30 | -0.07 | 0.01 | 0.31 | 0.07 | 0.01 | 0.33 | 0.05 | 0.00 | 0.51 | 0.06 | 0.00 | 0.40 | 0.09 | 0.01 | 0.20 |
|  | outer ring (SN) | -0.04 | 0.00 | 0.55 | 0.06 | 0.00 | 0.41 | 0.10 | 0.01 | 0.16 | -0.02 | 0.00 | 0.80 | 0.00 | 0.00 | 0.99 | 0.13 | 0.02 | 0.06 |
| sr = semipartial correlation coefficient, sr^2^ = semipartial correlation squared, IN = inferior nasal, IT = inferior temporal, ST = superior temporal, SN = superior nasal. | | | | | | | | | | | | | | | | | | | |
