# Supplementary material for: Influence of Clinical Factors and Magnification Correction on Normal Thickness Profiles of Macular Retinal Layers Using Optical Coherence Tomography
Source: PLoS One. 2016 Jan 27;11(1):e0147782. doi: 10.1371/journal.pone.0147782 (PMC4729678; doi:10.1371/journal.pone.0147782)
Supplement: S5 Table — (DOCX) [file pone.0147782.s005.docx]

| S5 Table. Semipartial correlation of various factors with the thickness of the outer retina in each analytical area | | | | | | | | | | | | | | | | | | | |  |
| --- | --- | --- | --- | --- | --- | --- | --- | --- | --- | --- | --- | --- | --- | --- | --- | --- | --- | --- | --- | --- |
| Factors | | Age | | | Gender | | | Eye laterality | | | Axial length | | | Corneal curvature | | | Signal strength index | | | |
|  | | sr | sr^2^ | p value | sr | sr^2^ | p value | sr | sr^2^ | p value | sr | sr^2^ | p value | sr | sr^2^ | p value | sr | sr^2^ | p value | |
| Magnification-uncorrected | total area | -0.06 | 0.00 | 0.34 | -0.32 | 0.10 | <0.0001 | -0.02 | 0.00 | 0.68 | -0.51 | 0.26 | <0.0001 | -0.05 | 0.00 | 0.36 | -0.08 | 0.01 | 0.18 | |
|  | center | 0.16 | 0.03 | 0.01 | -0.33 | 0.11 | <0.0001 | 0.04 | 0.00 | 0.56 | 0.10 | 0.01 | 0.14 | 0.00 | 0.00 | 0.99 | -0.07 | 0.01 | 0.26 | |
|  | inner ring (total) | -0.01 | 0.00 | 0.83 | -0.33 | 0.11 | <0.0001 | -0.02 | 0.00 | 0.80 | -0.42 | 0.18 | <0.0001 | -0.06 | 0.00 | 0.37 | -0.12 | 0.01 | 0.04 | |
|  | outer ring (total) | -0.08 | 0.01 | 0.18 | -0.29 | 0.09 | <0.0001 | -0.03 | 0.00 | 0.61 | -0.53 | 0.29 | <0.0001 | -0.05 | 0.00 | 0.36 | -0.06 | 0.00 | 0.28 | |
|  | inner ring (IN) | -0.01 | 0.00 | 0.91 | -0.34 | 0.12 | <0.0001 | -0.03 | 0.00 | 0.60 | -0.43 | 0.19 | <0.0001 | -0.07 | 0.01 | 0.25 | -0.13 | 0.02 | 0.03 | |
|  | inner ring (IT) | -0.03 | 0.00 | 0.66 | -0.33 | 0.11 | <0.0001 | -0.03 | 0.00 | 0.59 | -0.40 | 0.16 | <0.0001 | -0.03 | 0.00 | 0.61 | -0.13 | 0.02 | 0.04 | |
|  | inner ring (ST) | -0.01 | 0.00 | 0.83 | -0.31 | 0.10 | <0.0001 | 0.00 | 0.00 | 0.96 | -0.39 | 0.16 | <0.0001 | -0.05 | 0.00 | 0.44 | -0.10 | 0.01 | 0.10 | |
|  | inner ring (SN) | 0.00 | 0.00 | 0.94 | -0.33 | 0.11 | <0.0001 | 0.00 | 0.00 | 0.98 | -0.43 | 0.19 | <0.0001 | -0.07 | 0.00 | 0.29 | -0.11 | 0.01 | 0.08 | |
|  | outer ring (IN) | -0.07 | 0.00 | 0.24 | -0.26 | 0.07 | <0.0001 | -0.06 | 0.00 | 0.31 | -0.53 | 0.28 | <0.0001 | -0.06 | 0.00 | 0.31 | -0.06 | 0.00 | 0.33 | |
|  | outer ring (IT) | -0.08 | 0.01 | 0.16 | -0.30 | 0.09 | <0.0001 | -0.02 | 0.00 | 0.72 | -0.50 | 0.25 | <0.0001 | -0.02 | 0.00 | 0.70 | -0.07 | 0.01 | 0.23 | |
|  | outer ring (ST) | -0.08 | 0.01 | 0.21 | -0.29 | 0.09 | <0.0001 | -0.02 | 0.00 | 0.79 | -0.48 | 0.23 | <0.0001 | -0.04 | 0.00 | 0.51 | -0.07 | 0.01 | 0.24 | |
|  | outer ring (SN) | -0.07 | 0.01 | 0.22 | -0.28 | 0.08 | <0.0001 | -0.01 | 0.00 | 0.80 | -0.54 | 0.29 | <0.0001 | -0.08 | 0.01 | 0.18 | -0.04 | 0.00 | 0.45 | |
| Magnification-corrected | total area | -0.06 | 0.00 | 0.34 | -0.36 | 0.13 | <0.0001 | -0.03 | 0.00 | 0.67 | -0.25 | 0.06 | 0.0002 | -0.05 | 0.00 | 0.41 | -0.08 | 0.01 | 0.21 | |
|  | center | 0.16 | 0.03 | 0.02 | -0.32 | 0.11 | <0.0001 | 0.04 | 0.00 | 0.60 | 0.03 | 0.00 | 0.64 | 0.00 | 0.00 | 0.98 | -0.07 | 0.01 | 0.29 | |
|  | inner ring (total) | -0.01 | 0.00 | 0.85 | -0.36 | 0.13 | <0.0001 | -0.01 | 0.00 | 0.82 | -0.31 | 0.09 | <0.0001 | -0.06 | 0.00 | 0.35 | -0.12 | 0.02 | 0.06 | |
|  | outer ring (total) | -0.09 | 0.01 | 0.18 | -0.34 | 0.12 | <0.0001 | -0.03 | 0.00 | 0.60 | -0.23 | 0.05 | 0.0005 | -0.05 | 0.00 | 0.44 | -0.07 | 0.00 | 0.32 | |
|  | inner ring (IN) | -0.01 | 0.00 | 0.92 | -0.36 | 0.13 | <0.0001 | -0.03 | 0.00 | 0.63 | -0.30 | 0.09 | <0.0001 | -0.07 | 0.01 | 0.26 | -0.14 | 0.02 | 0.03 | |
|  | inner ring (IT) | -0.03 | 0.00 | 0.66 | -0.35 | 0.12 | <0.0001 | -0.03 | 0.00 | 0.64 | -0.28 | 0.08 | <0.0001 | -0.04 | 0.00 | 0.56 | -0.13 | 0.02 | 0.05 | |
|  | inner ring (ST) | -0.01 | 0.00 | 0.88 | -0.33 | 0.11 | <0.0001 | 0.01 | 0.00 | 0.94 | -0.29 | 0.09 | <0.0001 | -0.06 | 0.00 | 0.40 | -0.11 | 0.01 | 0.11 | |
|  | inner ring (SN) | 0.00 | 0.00 | 0.99 | -0.35 | 0.12 | <0.0001 | 0.00 | 0.00 | 0.98 | -0.33 | 0.11 | <0.0001 | -0.07 | 0.01 | 0.27 | -0.11 | 0.01 | 0.10 | |
|  | outer ring (IN) | -0.08 | 0.01 | 0.22 | -0.31 | 0.10 | <0.0001 | -0.07 | 0.01 | 0.28 | -0.24 | 0.06 | 0.0003 | -0.06 | 0.00 | 0.35 | -0.06 | 0.00 | 0.37 | |
|  | outer ring (IT) | -0.09 | 0.01 | 0.16 | -0.35 | 0.12 | <0.0001 | -0.03 | 0.00 | 0.69 | -0.19 | 0.03 | 0.01 | -0.01 | 0.00 | 0.84 | -0.08 | 0.01 | 0.25 | |
|  | outer ring (ST) | -0.09 | 0.01 | 0.20 | -0.32 | 0.10 | <0.0001 | -0.02 | 0.00 | 0.81 | -0.19 | 0.04 | 0.01 | -0.04 | 0.00 | 0.60 | -0.07 | 0.01 | 0.27 | |
|  | outer ring (SN) | -0.08 | 0.01 | 0.23 | -0.32 | 0.10 | <0.0001 | -0.02 | 0.00 | 0.82 | -0.27 | 0.07 | 0.0001 | -0.08 | 0.01 | 0.22 | -0.04 | 0.00 | 0.53 | |
| sr = semipartial correlation coefficient, sr^2^ = semipartial correlation squared, IN = inferior nasal, IT = inferior temporal, ST = superior temporal, SN = superior nasal. | | | | | | | | | | | | | | | | | | | |  |
