# Supplementary material for: Influence of Clinical Factors and Magnification Correction on Normal Thickness Profiles of Macular Retinal Layers Using Optical Coherence Tomography
Source: PLoS One. 2016 Jan 27;11(1):e0147782. doi: 10.1371/journal.pone.0147782 (PMC4729678; doi:10.1371/journal.pone.0147782)
Supplement: S6 Table — (DOCX) [file pone.0147782.s006.docx]

| S6 Table. Between-gender differences in the thickness of retinal layers in each analytical area. | | | | | | | | | | | | | | | | | | | | |
| --- | --- | --- | --- | --- | --- | --- | --- | --- | --- | --- | --- | --- | --- | --- | --- | --- | --- | --- | --- | --- |
| Retinal layers | RNFL | | | | GCLIPL | | | | GCC | | | | Total retina | | | | Outer retina | | | |
| Magnification correction | - | | + | | - | | + | | - | | + | | - | | + | | - | | + | |
| Gender | Male | Female | Male | Female | Male | Female | Male | Female | Male | Female | Male | Female | Male | Female | Male | Female | Male | Female | Male | Female |
| Total area | 34.8 ± 2.9 | 34.8 ± 3.2 | 33.7± 2.4 | 34.1 ± 2.5 | 68.6 ± 5.7 | 68.7 ± 5.5 | 71.2 ± 4.9 | 70.0 ± 4.5 | 103.4 ± 6.7 | 103.5 ± 6.4 | 104.9 ± 6.4 | 104.1 ± 6.1 | 305.0 ± 12.2 | 300.8 ± 11.8 | 307.4 ± 11.3* | 301.7 ± 10.8* | 200.1 ± 8.3* | 196.5 ± 8.6* | 202.5 ± 7.3* | 197.6 ± 7.4* |
| Center | 6.3 ± 5.2 | 4.9 ± 5.0 | 5.6 ± 5.0 | 4.7 ± 4.9 | 43.4 ± 7.8* | 40.0 ± 6.4* | 40.6 ± 8.4 | 38.6 ± 7.2 | 49.6 ± 8.3* | 44.8 ± 7.4* | 46.2 ± 7.6* | 43.3 ± 6.6* | 269.2 ± 15.1* | 258.4 ± 15.3* | 268.7 ± 15.6* | 258.4 ± 15.6* | 223.1 ± 10.0* | 215.4 ± 11.1* | 222.5 ± 10.3* | 215.1 ± 11.3* |
| Inner ring (total) | 26.6 ± 2.8* | 25.0 ± 3.5* | 25.6 ± 2.3* | 24.5 ± 2.8* | 93.2 ± 6.4 | 91.2 ± 6.5 | 93.4 ± 6.6 | 91.2 ± 6.5 | 119.8 ± 6.6* | 116.1 ± 7.6* | 119.0 ± 6.6* | 115.7 ± 7.4* | 340.2 ± 12.7 | 331.9 ± 12.9 | 341.7 ± 12.5* | 333.0 ± 12.8* | 221.3 ± 9.0* | 216.8 ± 9.2* | 222.7 ± 8.5* | 217.4 ± 8.6* |
| Outer ring (total) | 38.3 ± 3.3 | 38.8 ± 3.4 | 37.1 ± 2.9 | 38.0 ± 2.8 | 62.2 ± 6.2 | 63.1 ± 6.0 | 65.8 ± 4.9 | 64.8 ± 4.5 | 100.6 ± 7.5* | 101.9 ± 6.8 | 102.9 ± 6.9 | 102.9 ± 6.3 | 265.9 ± 12.8 | 293.2± 12.2 | 298.7 ± 11.8* | 294.0 ± 10.7* | 192.9 ± 8.5 | 189.8 ± 8.7 | 195.8 ± 7.2* | 191.1 ± 7.3* |
| Inner ring (IN) | 28.9 ± 3.1* | 27.1 ± 4.2* | 27.6 ± 2.8* | 26.4 ± 3.2* | 93.3 ± 6.6 | 91.1 ± 6.3 | 93.4 ± 6.9 | 91.1 ± 6.3 | 122.3 ± 7.0* | 118.2 ± 7.8* | 121.0 ± 7.1* | 117.5 ± 7.6* | 342.3 ± 13.5* | 333.3 ± 12.9* | 344.1 ± 13.2* | 334.7 ± 12.8* | 221.4 ± 9.7* | 216.4 ± 9.7* | 223.0 ± 9.1* | 217.2 ± 9.0* |
| Inner ring (IT) | 25.0 ± 3.2* | 23.2 ± 4.2* | 24.4 ± 2.9 | 23.0 ± 3.7 | 93.2 ± 6.8 | 90.9 ± 7.1 | 93.5 ± 6.9 | 91.0 ± 7.0 | 118.2 ± 6.8* | 114.1 ± 7.7* | 117.8 ± 6.9* | 114.0 ± 7.6* | 335.8 ± 12.8* | 327.0 ± 12.8* | 337.2 ± 12.6* | 328.0 ± 12.7* | 218.1 ± 8.9* | 213.4 ± 9.3* | 219.4 ± 8.5* | 214.0 ± 8.8* |
| Inner ring (ST) | 24.7 ± 2.9 | 23.6 ± 3.4 | 24.1 ± 2.6 | 23.3 ± 3.0 | 92.0 ± 6.6 | 90.0 ± 6.9 | 92.4 ± 6.6 | 90.2 ± 6.9 | 116.7 ± 6.5* | 113.6 ± 7.6* | 116.4 ± 6.5* | 113.5 ± 7.4* | 336.9 ± 12.5* | 329.6 ± 12.9* | 338.1 ± 12.3* | 330.3 ± 12.8* | 220.6 ± 8.9* | 216.3 ± 9.2* | 221.7 ± 8.5* | 216.8 ± 8.8* |
| Inner ring (SN) | 27.7 ± 3.3* | 26.0 ± 4.0* | 26.3 ± 2.7 | 25.2 ± 3.1 | 94.3 ± 6.7 | 92.6 ± 6.8 | 94.3 ± 7.0 | 92.5 ± 6.8 | 122.0 ± 7.1* | 118.6 ± 8.4* | 120.6 ± 7.1 | 117.7 ± 8.1 | 345.8 ± 13.4* | 338.0 ± 14.0* | 347.3 ± 13.2* | 339.2 ± 13.9* | 225.4 ± 9.3* | 220.9 ± 9.6* | 226.7 ± 8.8* | 221.5 ± 9.0* |
| Outer ring (IN) | 49.2 ± 6.0 | 49.7 ± 6.0 | 46.3 ± 5.1* | 47.9 ± 4.4* | 60.9 ± 6.1 | 62.9 ± 6.2 | 64.5 ± 4.8 | 64.5 ± 4.6 | 110.1 ± 9.1 | 112.6 ± 7.7 | 110.8 ± 8.5 | 112.5 ± 7.1 | 300.2 ± 15.0 | 299.3 ± 13.3 | 303.3 ± 13.9 | 300.5 ± 12.0 | 189.4 ± 9.4 | 186.7 ± 9.4 | 192.6 ± 8.0* | 188.1 ± 7.8* |
| Outer ring (IT) | 28.7 ± 2.7 | 28.7 ± 2.9 | 28.6 ± 2.6 | 28.5 ± 2.8 | 61.4 ± 6.5 | 61.7 ± 6.4 | 64.9 ± 5.5 | 63.4 ± 4.9 | 90.1 ± 7.0 | 90.3 ± 7.2 | 93.5 ± 6.3 | 92.0 ± 6.2 | 282.6 ± 12.1 | 278.4 ± 12.2 | 285.4 ± 11.1* | 278.8 ± 10.8* | 189.0 ± 8.4* | 185.5 ± 8.6* | 191.9 ± 7.3* | 186.8 ± 7.3* |
| Outer ring (ST) | 27.7 ± 2.6 | 28.1 ± 2.7 | 27.8 ± 2.6 | 28.1 ± 2.7 | 62.0 ± 6.9 | 62.2 ± 6.2 | 65.4 ± 6.1 | 63.8 ± 5.2 | 89.6 ± 7.6 | 90.3 ± 7.5 | 93.2 ± 6.9 | 91.9 ± 6.8 | 288.5 ± 12.6 | 284.7 ± 12.5 | 290.9 ± 11.8* | 285.0 ± 11.3* | 195.2 ± 8.1 | 191.9 ± 8.5 | 197.8 ± 7.3* | 193.1 ± 7.5* |
| Outer ring (SN) | 47.8 ± 5.1 | 48.8 ± 4.9 | 45.7 ± 4.3* | 47.5 ± 3.9* | 64.7 ± 6.4 | 65.8 ± 6.5 | 68.3 ± 5.0 | 67.6 ± 5.1 | 112.5 ± 8.4 | 114.6 ± 7.6 | 114.0 ± 7.9 | 115.1 ± 7.4 | 312.0 ± 14.1 | 310.5 ± 13.5 | 315.0 ± 12.9 | 311.6 ± 12.1 | 198.0 ± 9.2 | 195.0 ± 9.9 | 201.0 ± 7.8* | 196.5 ± 8.2* |
| RNFL = retinal nerve fiber layer, GCLIPL = ganglion cell layer plus inner plexiform layer, GCC = ganglion cell complex, IN = inferior nasal, IT = inferior temporal, ST = superior temporal, SN = superior nasal.  *P <0.05 after Bonferroni correction (unpaired t-test). Data are shown as mean ± standard deviation (µm). | | | | | | | | | | | | | | | | | | | | |
